# Long Non-coding RNA H19 Suppression Protects the Endothelium Against Hyperglycemic-Induced Inflammation via Inhibiting Expression of *miR-29b* Target Gene Vascular Endothelial Growth Factor a Through Activation of the Protein Kinase B/Endothelial Nitric Oxide Synthase Pathway

**DOI:** 10.3389/fcell.2019.00263

**Published:** 2019-11-01

**Authors:** Xiao-wen Cheng, Zhen-fei Chen, Yu-feng Wan, Qing Zhou, Hua Wang, Hua-qing Zhu

**Affiliations:** ^1^Department of Clinical Laboratory, The First Affiliated Hospital, Anhui Medical University, Hefei, China; ^2^Laboratory of Molecular Biology, Department of Biochemistry, Anhui Medical University, Hefei, China; ^3^Department of Vasculocardiology, Hefei Hospital Affiliated to Anhui Medical University, Hefei, China; ^4^Department of Otolaryngology, The Affiliated Chaohu Hospital, Anhui Medical University, Hefei, China; ^5^Department of Oncology, The First Affiliated Hospital, Institute for Liver Disease, Anhui Medical University, Hefei, China

**Keywords:** diabetes, *H19*, inflammation, miRNA-29b, vascular endothelial growth factor A

## Abstract

It has been shown that non-coding RNAs (ncRNAs) play an important regulatory role in pathophysiological processes involving inflammation. The vascular endothelial growth factor A (*VEGFA*) gene also participates in the inflammatory process. However, the relationships between ncRNAs and VEGFA are currently unclear. Here, this study was designed to determine the relationship between long non-coding RNA (lncRNA) H19, mircoRNA29b (miR-29b), and VEGFA in the development of diabetes mellitus (DM). We demonstrate that H19 is upregulated and miR-29b downregulated in individuals with DM and directly binds miR-29b. VEGFA is the target of miR-29b in the vascular endothelium of individuals with DM. We found that positive modulation of miR29b and inhibition of H19 and VEGFA significantly attenuates high glucose-induced endothelial inflammation and oxidative stress. We also found that the protein kinase B/endothelial nitric oxide synthase (AKT/eNOS) signal pathway in endothelial cells is activated through regulation of miR29b and H19 endogenous RNAs. We conclude that H19 suppression protects the endothelium against high glucose-induced inflammation and oxidative stress in endothelial cells by upregulation of miR-29b and downregulation of *VEGFA* through AKT/eNOS signal pathway activation. These results suggest a novel link between dysregulated ncRNA expression, inflammation, and the signaling pathway in the vascular endothelium of individuals with DM, indicating a promising strategy for preventing cardiovascular disease in such individuals.

## Introduction

Impaired vascular remodeling is considered to be involved in the development of cardiovascular disease (CVD) (García-Redondo et al., 2016). It is generally accepted that there is a relationship between CVD and vascular inflammation (Haybar et al., 2019). Vascular inflammation also contributes to the progression of more complex diseases such as atherosclerosis (AS) and diabetes mellitus (DM). There is strong evidence that DM independently increases the risk of CVD and that pro-inflammatory components play a key role in its development (Fishman et al., 2018; Yuan et al., 2019). Brownlee (2001) have proposed that hyperglycemia plays a role in promoting overproduction of reactive oxygen species (ROS) by the mitochondrial electron transport chain and that prolonged production of mitochondrial superoxide contributes to development of diabetes-related vascular damage. ROS are generated in the vascular wall by nicotinamide adenine dinucleotide phosphate (NADPH) oxidase, xanthine oxidase, the mitochondrial electron transport chain, and uncoupled endothelial nitric oxide synthase (eNOS). Therefore, it would be useful to identify new inflammatory factors associated with the development of CVD. In this study, we aimed to investigate whether vascular epithelial growth factor A (VEGFA), a member of the VEGF family that is considered to be pro-inflammatory cytokines, is involved in the pathogenesis of CVD in individuals with DM.

Sequencing experiments have shown that more than 90% of the genome is transcribed into non-coding RNA (ncRNA). ncRNA includes microRNA (miRNA; 18–24 nucleotides) and long ncRNA (lncRNA; >200 nucleotides). miRNAs and lncRNAs have different functions. miRNAs bind to the 3′-UTR of target genes to mediate translational repression, thereby altering the biology of diverse disease states. Meanwhile, lncRNAs have emerged as powerful biological regulators that act by modulating numerous cellular processes. lncRNAs can act as miRNA sponges and inhibit miRNA expression. Numerous studies have suggested that ncRNAs play a key role in regulating pathophysiological processes involved in CVD (Hung et al., 2018; Simion et al., 2019). H19, a paternally imprinted and maternally expressed gene, produces a 2.3-kb spliced, capped, and polyadenylated lncRNA (Yoshimura et al., 2018). H19 is up-regulated in AS and associated with its progression but the underlying regulatory mechanisms have not yet been conclusively established (Bitarafan et al., 2019). H19 expression is chronically increased in individuals with DM but the biological significance of this is not yet understood (Zhang N. et al., 2018). Recent studies have shown that H19 upregulates VEGFA to enhance the survival and angiogenic capacity of mesenchymal stem cells through miR-199a-5p inhibition. This also mediates the antiapoptotic effect of hypoxic postconditioning against hypoxia-reoxygenation-induced injury in aged cardiomyocytes by inhibiting miR-29b-3p expression (Hou et al., 2018; Zhang et al., 2019). In the present study, we explored the interaction between VEGFA and H19 during hyperglycemia-induced inflammation and investigated whether miR-29b is involved in this interaction.

## Materials and Methods

### Patients

The study cohort comprised 30 patients with DM and 30 healthy individuals all of whom attended the First Affiliated Hospital of Anhui Medical University. DM was diagnosed following the 1999 World Health Organization guidelines and classification. Peripheral blood (10 ml; EDTA anticoagulation) was obtained from each participant. Blood samples were centrifuged at 1500 *g* for 10 min to collect plasma. Plasma samples were aliquoted into Eppendorf tubes (500 μl in each), and all samples were stored at −80°C until further analysis. The study protocols were approved by the Ethics Committee of Anhui Medical University. All patients provided written informed consent.

### Cell Culture

Human umbilical vein endothelial cells (HUVECs) obtained from the American Type Culture Collection (Manassas, VA, United States) were cultured in Dulbecco’s Modified Eagles Media (Thermo Fisher Scientific, Beijing, China) supplemented with 10% fetal bovine serum and 100 IU/ml penicillin/streptomycin (Invitrogen, Carlsbad, CA, United States). Cells were maintained at 37°C with 5% CO_2_.

### Cell Transfection

MiR-29b mimics and inhibitors (GenePharm Co., Ltd., Shanghai, China) were used to upregulate or downregulate miR-29b expression. Cells were transfected with VEGFA-siRNA (GenePharm), H19-shRNA (GenePharm), or miR-29b mimics, inhibitors, or controls (GenePharm) using Lipofectamine 2000 (Invitrogen; Thermo Fisher Scientific, Inc., Waltham, MA, United States) according to the manufacturer’s protocol. A scrambled oligonucleotide (GenePharm) served as a control. Changes in RNA expression were determined by qRT-PCR 24 h after transfection, and changes in protein expression were measured by western blotting 48 h after transfection.

### Determination of ROS, Tumor Necrosis Factor-Alpha (TNF-α), and NADPH

Reactive oxygen species production was assessed following the method described by Zhu et al. (2009). The proteins obtained from the HUVECs were incubated with 20 μM 2′,7′-dichlorofluorescin diacetate at 37°C for 3 h. Fluorescence was measured by spectrofluorometry at an excitation of 488 nm and an emission of 525 nm. TNF-α titers were determined by enzyme-linked immunosorbent assay (eBioscience, San Diego, CA, United States). Lucigenin-enhanced chemiluminescence was used to evaluate NADPH oxidase activity in cell lysates using a multilabel counter (VICTOR3; PerkinElmer-Wallac, Waltham, MA, United States) (Li et al., 2009). Light signals were detected every 5 s. NADPH oxidase activity was calculated and is presented as counts per second.

### Western Blotting

Cells were then harvested and lysed with 1 × sodium dodecyl sulfate (SDS) lysis buffer containing 50 mM Tris–HCl (pH 6.8), 10% glycerol, and 2% SDS. Cell lysates were boiled for 10 min then centrifuged at 12,000 *g* for 15 min at room temperature. Samples were separated by 12% SDS-PAGE and transferred to a polyvinylidene difluoride membrane (GE Healthcare, Piscataway, NJ, United States). The membranes were blocked in 5% bovine serum albumen for 2 h, followed by a 4°C overnight incubation with primary antibodies. Primary antibodies were detected with corresponding horseradish peroxidase-conjugated secondary antibodies (Zhongshan Jinqiao, Beijing, China) coupled with enhanced chemiluminescence reagents (Engreen, Beijing, China).

### Luciferase Assay

The H19 and VEGFA 3′-UTR regions, containing potential miR-29b binding sites, were predicted using TargetScan version 7.1^1^. The predicted 3′-UTR fragments were amplified by PCR. Mutants were then constructed by introducing point mutations into the seed binding site for miR-29b. The wild type and mutant fragments (wt-Luc-H19 and wt-Luc-VEGFA, and mu-Luc-H19 and mu-Luc-VEGFA) were subcloned into the psiCHECK2 vector (Promega Corporation, United States), downstream of the renilla luciferase gene. The vector also contains the firefly luciferase gene. Cells were seeded in 24-well plates and cotransfected with wild-type or mutated luciferase constructs along with miR-29b mimics, miR-29b inhibitors, or controls. The Dual Luciferase Reporter Assay System (Promega) was used 48 h after transfection following the manufacturer’s protocol. The relative luciferase activity was calculated using the ratio of firefly luciferase activity to renilla luciferase activity.

### RNA Immunoprecipitation (RIP)

We assessed the direct interaction between miR-29b and lncRNA H19 by Argonaute 2 (Ago2)-RNA immunoprecipitation (Ago2-RIP). Anti-Ago2 (Sigma-Aldrich, United States), or control anti-IgG and Dynabeads Protein G (Invitrogen, United States) were incubated at 4°C with rotation a day prior to the experiment. Complete RIP lysis buffer, containing protease inhibitor, phosphatase inhibitor (Roche, Switzerland), and RNase inhibitor (Invitrogen, United States), was used to lyse cells. RNA in Ago2-RIP materials was washed several times with PEB buffer and treated with DNase I and Proteinase K (Promega). RNA was isolated with Trizol (Invitrogen) and precipitated with absolute ethanol overnight at −20°C. After the removal of the proteins and beads, RT-qPCR analysis of the purified RNA, and lncRNA H19 enrichment in Ago2-RIP, was performed.

### Reverse Transcription-Quantitative Polymerase Chain Reaction (RT-qPCR)

Total RNA from mouse tissues and GC-1 cells was extracted using TRIzol reagent following the manufacturer’s instructions (Invitrogen, Carlsbad, CA, United States). Isolated total RNA (1 μg) was converted to complementary DNA (cDNA) using a First-Strand cDNA Synthesis Kit (Toyobo, Osaka, Japan). Power SYBR green master mix (Applied Biosystems, Foster City, CA, United States) was added to the cDNA samples, which were then subjected to qRT-PCR using a StepOne Real Time PCR system. *GAPDH* and U6 were used as endogenous controls for mRNAs/lncRNAs and for miRNAs, respectively.

The amplification results were calculated using the 2 (−ΔΔCt) method. The PCR primers used were: H19, forward 5′-A TCGGTGCCTCAGCGTTCGG-3′, reverse 5′-CTGTCCTCGC CGTCACACCG-3′; *VEGFA*, forward 5′-TGGCTCACTGGCT TGCTCTA-3′, and reverse 5′-ATCCAACTGCACCGTCACAG-3′; *GAPDH*, forward 5′-GGTGGTCTCCTCTGACTTCAA-3′, and reverse 5′-GTTGCTGTAGCCAAATTCGTTGT-3′; miR-29b, 5′UAGCACCAUUUGAAAUCAGUGUU-3′; and U6, 5′-CGCTTCGGCAGCACATATACTAAAATTGGAAC-3′.

### Statistical Analysis

Statistical analysis was performed using SPSS software version 20.0. The data were subjected to one-way ANOVA and parametric *t*-testing and are presented as the mean ± SD. The tests were performed at least in triplicate. *P* < 0.05 was considered to denote statistical significance.

## Results

### Relationships Between H19, miR-29b, and VEGFA

**FIGURE 1:**
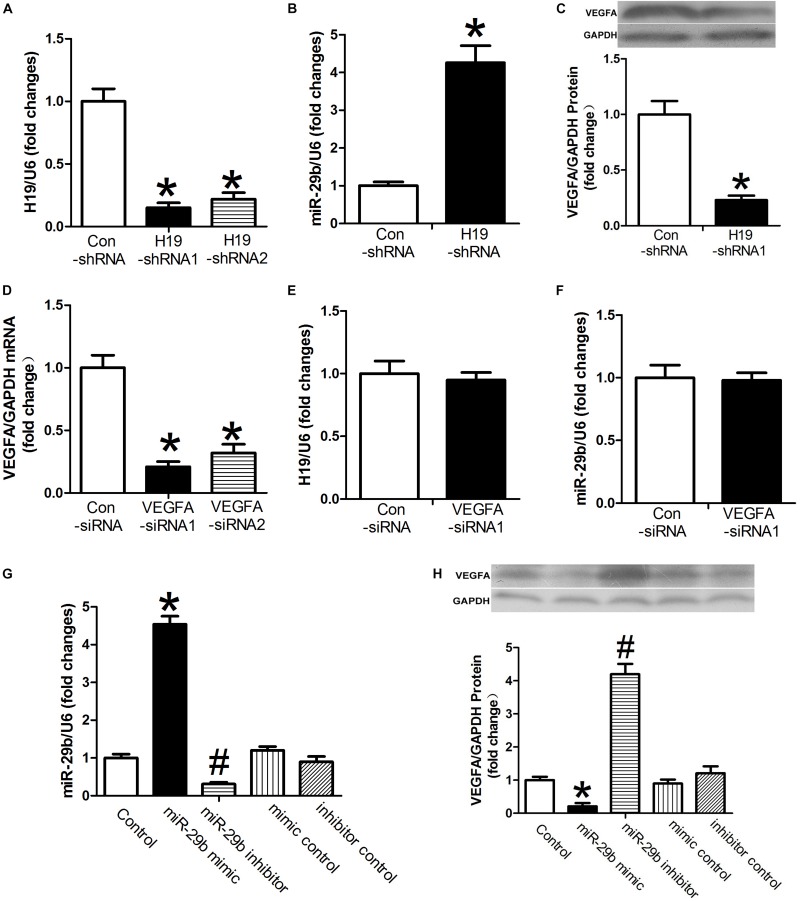
The relationships between H19, miR-29b, and VEGFA. **(A)** The inhibitory effects of H19-shRNA on H19 mRNA expression were determined by RT-PCR. **(B,C)** Human umbilical vein endothelial cells (HUVECs) were transfected with H19-shRNA1. miR29b and VEGFA expression were measured by RT-PCR after 24 h and by western blot after 48 h. **(D)** RT-PCR showed *VEGFA* mRNA expression is downregulated after exposure to VEGFA-siRNA1. **(E,F)** H19 and miR-29b expression was measured following 24 h of exposure to VEGFA-siRNA1. **(G,H)** miR29b and VEGFA expression was measured in the presence of miR-29b mimics. ^∗^*P* < 0.05 vs. control group. ^#^*P* < 0.05 vs. miR-29b inhibitor control group.

Expression of H19 was significantly downregulated after treatment with either H19-shRNA1 or H19-shRNA2 (*P* < 0.05, Figure 1A). In cases of H19 downregulation, expression of miR-29b was significantly upregulated (*P* < 0.05, Figure 1B). Additionally, after H19 downregulation, VEGFA protein expression was also significantly reduced (*P* < 0.05, Figure 1C). VEGFA-siRNA1 and VEGFA-siRNA2 transfection significantly downregulated *VEGFA* expression, especially VEGFA-siRNA1 (*P* < 0.05, Figure 1D). VEGFA-siRNA1 transfection did not result in significant changes in H19 or miR-29b expression (*P* > 0.05, Figures 1E,F). However, *VEGFA* expression was significantly decreased in the presence of miR-29b mimics (Figures 1G,H), implying a potential association between H19, miR-29b, and VEGFA.

### Expression of H19 and miR-29b in Patients With DM and Glucose-Treated HUVECs

**FIGURE 2:**
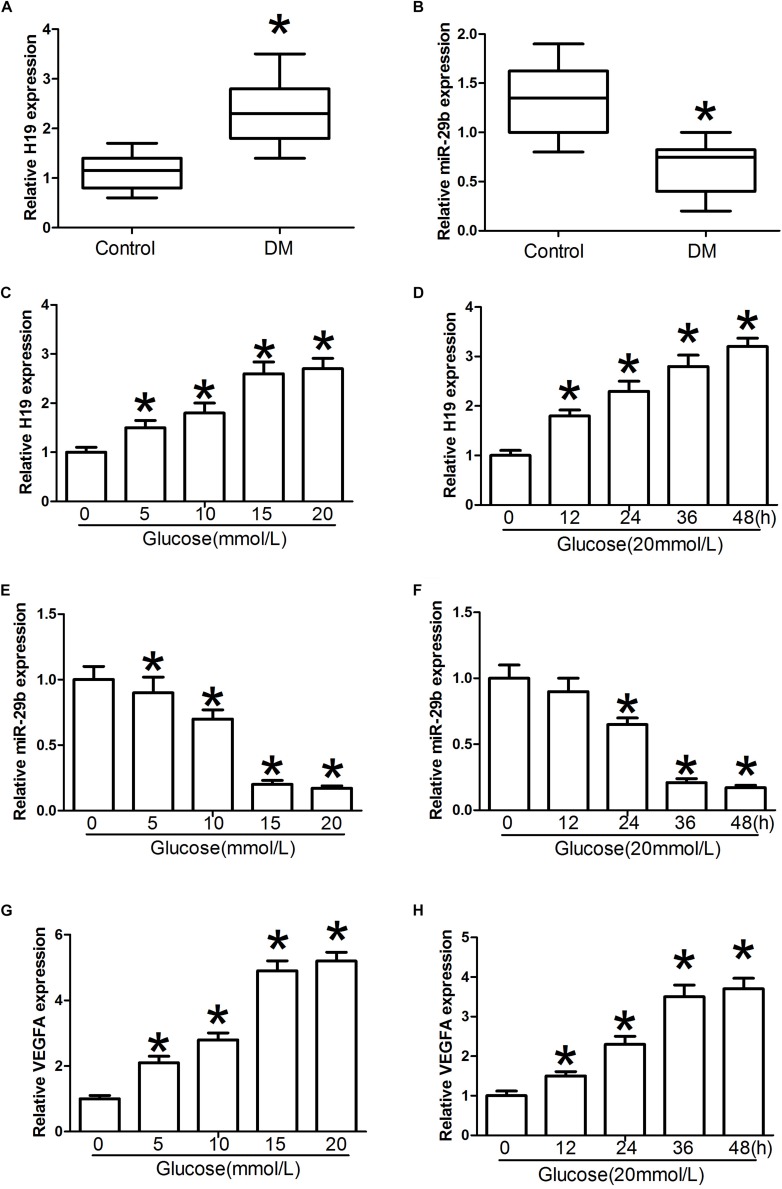
H19 and miR-29b expression in individuals with diabetes mellitus (DM) and in glucose-treated HUVECs. **(A,B)** Circulating lncRNA H19 and miR-29b levels were measured by RT-PCR. **(C–H)** Relative H19, miR-29b, and VEGFA expression about 24 h after exposure to various concentrations of glucose (0, 5, 10, 15, and 20 mmol/l) or treatment with glucose (20 mmol/l) for the indicated times. ^∗^*P* < 0.05 vs. control group.

H19 levels were significantly higher in blood samples obtained from individuals with DM than in the control individuals (*P* < 0.05, Figure 2A). Furthermore, relative miR-29b expression in individuals with DM was significantly weaker than that observed in the control individuals (*P* < 0.05, Figure 2B). Treatment with glucose in various doses and timings was associated with significant dose- and time-dependent H19 upregulation (*P* < 0.05, Figures 2C,D). In contrast, expression of miR-29b was significantly downregulated after administration of glucose in a dose- and time-dependent manner (*P* < 0.05, Figures 2E,F). Treatment with glucose in different doses and timings was also associated with significant dose- and time-dependent increase in VEGFA expression compared with the control group (*P* < 0.05, Figures 2G,H).

### H19 Binds miR-29b Directly and VEGFA Is a Target of miR-29

**FIGURE 3:**
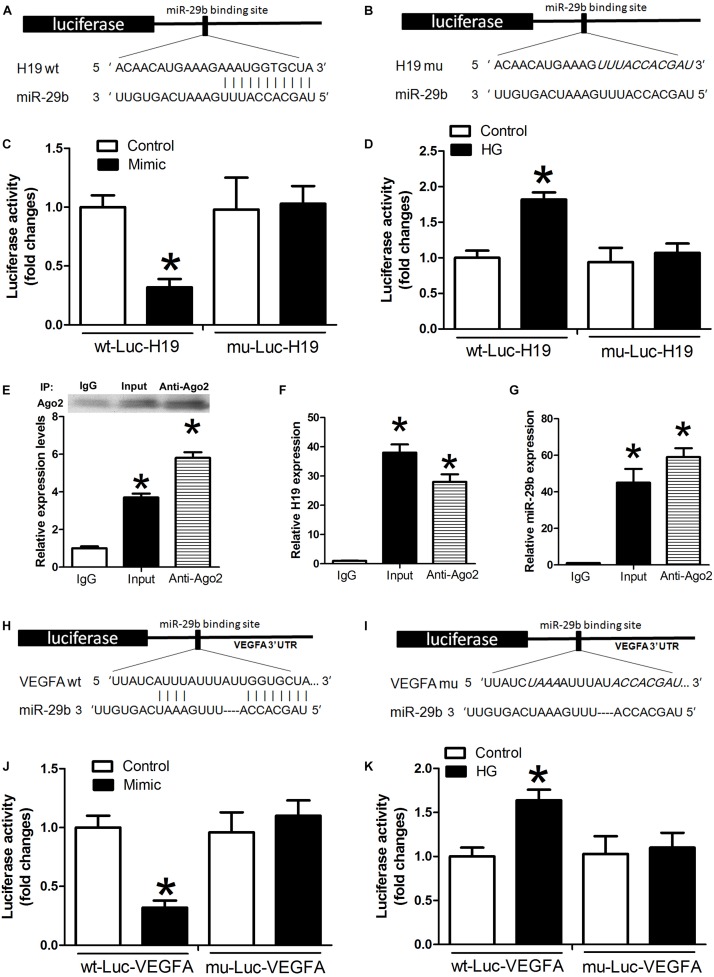
Potential miR-29b binding sites in H19 and VEGFA. **(A,B)** H19 3′-UTR, wild type and mutated miR-29b binding sites. miR-29b mimic and luciferase constructs were co-transfected into HUVECs **(C)** and HUVECs exposed to high glucose concentrations **(D)**. Cellular lysates from HUVECs were used for RNA immunoprecipitation with an Ago2 antibody. Ago2 protein levels were measured by western blotting **(E)**, the amount of H19 **(F)**, and miR-29b **(G)** in the immunoprecipitate was measured by RT-PCR. Wild type **(H)** and mutated miR-29b **(I)** binding sites in the *VEGFA* 3’-UTR are shown in the upper panel. **(J,K)** Luciferase activity of wild type and mutant Luc VEGFA. ^∗^*P* < 0.05 vs. control group. Mimic:miR29b mimic.

Luciferase assay results indicated that miR-29b mimics induced decreases in H19 luciferase activity. However, this effect was not observed with the H19 mutants (*P* < 0.05, Figures 3A,C), and there were no statistically significant differences between the miR-29b group and controls (*P* > 0.05, Figures 3A,C). Treatment with high glucose levels induced a significant increase in wild type H19 luciferase activity, and this effect was reduced after mutation at certain H19 sites (Figures 3B,D). An RNA immunoprecipitation (RIP) experiment was performed to investigate whether H19 and miR-29b are components of the RNA-induced silencing complex. An Ago2 antibody was used to precipitate Ago2 protein from cultured cells (Figure 3E). mRNA expression of both H19 and miR-29b was significantly enriched in the immunoprecipitates (Figures 3F,G), indicating that there is direct binding between H19 and miR-29b. A luciferase test was performed to identify potential VEGFA miR-29b binding sites. Our results show that miR-29b mimics induce decreases in VEGFA luciferase activity. However, this effect was not observed in the mutant (*P* < 0.05, Figures 3H,J) and there were no statistically significant differences between the miR-29b group and the control in VEGFA mutants (*P* > 0.05, Figures 3F,G). In the wild type, high glucose treatment induced significant increases in luciferase activity, while this effect was reduced after mutation of certain VEGFA sites (Figures 3I,K).

### Determination of TNF-α Expression, ROS Production, and NADPH Oxidase Activity in HUVECs Treated With High Concentrations of Glucose

**FIGURE 4:**
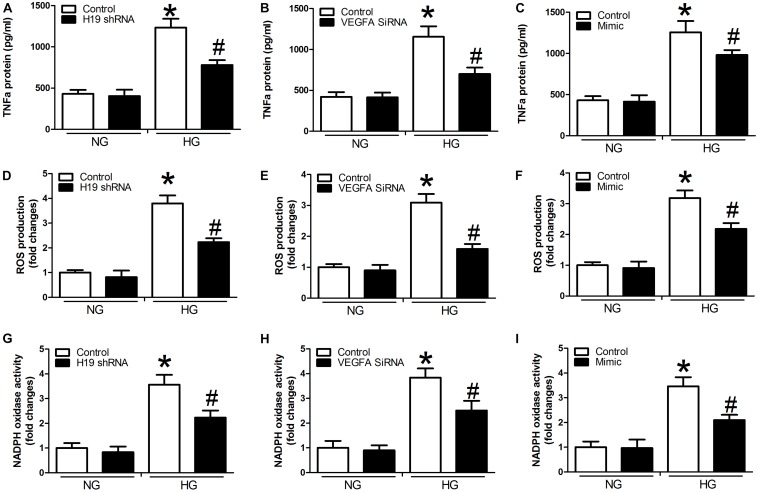
H19, VEGFA, and miR-29b regulate the expression of TNF-α, ROS production, and NADPH oxidase activity under normal glucose (NG: 5 mM) or high glucose (HG: 20 mM) conditions. **(A–C)** TNF-α protein expression was determined after treatment with H19 shRNA, VEGFA siRNA, and miR-29b mimic, as was ROS production **(D–F)**, and NADPH oxidase activity **(G–I)**. ^∗^*P* < 0.05, compared with control group in the presence of NG. ^#^*P* < 0.05, compared with control group in the presence of HG. mimic, miR-29b mimic.

**FIGURE 5:**
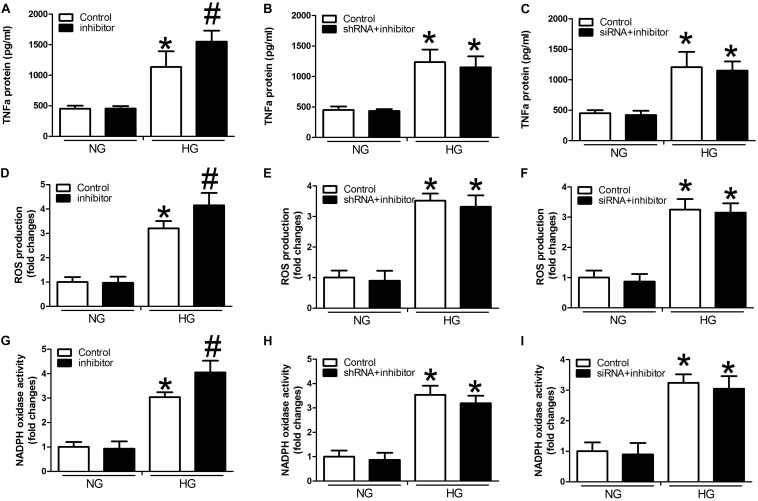
miR-29b inhibitor alone and in combination with H19 shRNA and VEGFA siRNA can regulate the expression of TNF-α, ROS production, and NADPH oxidase activity. Cells were transfected with miR29b inhibitor, or H19-shRNA + miR-29b inhibitor, or VEGFA-siRNA + miR-29b inhibitor and were cultured under normal glucose (NG: 5 mM) or high glucose (HG: 20 mM) conditions. Decreased expression of TNF-α **(A–C)**, ROS **(D–F)**, and NADPH **(G–I)** oxidase activity observed in response to H19 silencing or VEGFA silencing was suppressed by miR-29b inhibitors. ^∗^*P* < 0.05, compared with control group in the presence of NG. ^#^*P* < 0.05 vs. miRNA-29b inhibitor group under normal glucose condition (NG, 5 mM). Inhibitor:miRNA-29b inhibitor; shRNA:H19 shRNA; siRNA:VEGFA siRNA.

Compared with controls, we detected significant activation of TNF-α expression, ROS production, and NADPH oxidase activity in the presence of high glucose concentrations in the miR-29b inhibitor alone group (*P* < 0.05, Figures 4A,D,G). We also observed obvious inhibition of TNF-α expression, ROS production, and NADPH oxidase activity in the H19 shRNA, VEGFA siRNA, and miR-29b mimic groups in the presence of high glucose concentrations (*P* < 0.05, Figures 4B,C,E,F,H,I). In addition, miR-29b inhibitor treatment reversed the effects of H19 shRNA and VEGFA siRNA on TNF-α expression, ROS production, and NADPH oxidase activity in the presence of high glucose concentrations (*P* < 0.05, Figure 5).

### Role of H19/miR-29b/VEGFA in the AKT-eNOS Pathway

**FIGURE 6:**
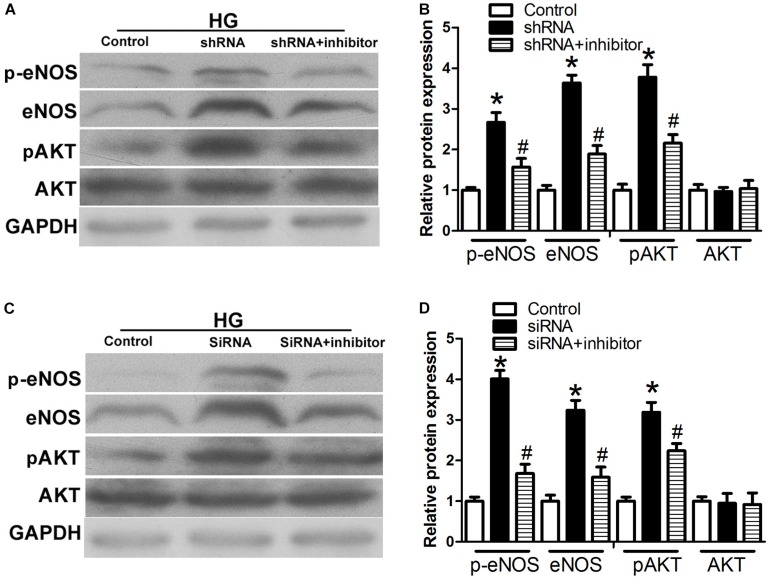
The H19/miR-29b/VEGFA pathway is associated with the AKT–NOS pathway. **(A,B)** Expression of p-eNOS, eNOS, and p-AKT in HUVECs after treatment with H19 shRNA and H19 shRNA + miR-29b inhibitor. ^∗^*P* < 0.05 vs. control group. ^#^*P* < 0.05 vs. shRNA group. **(C,D)** Expression of p-eNOS, eNOS, and p-AKT in HUVECs after treatment with *VEGFA* siRNA and *VEGFA* siRNA + miR-29b inhibitor. ^∗^*P* < 0.05 vs. control group. ^#^*P* < 0.05 vs. siRNA group (HG, 20 mM). Inhibitor:miRNA-29b inhibitor; shRNA:H19 shRNA.

To test whether the observed changes in endogenous RNAs and inflammatory factors are related to the AKT-eNOS pathway, we assessed the expression of p-eNOS, eNOS, p-AKT, and AKT. p-eNOS, eNOS, and p-AKT expression levels were higher in the H19 shRNA group than those observed in the control group (*P* < 0.05, Figures 6A,B). However, this effect was suppressed in the H19shRNA + miR29b inhibitor group (*P* < 0.05, Figures 6A,B). Expression of p-eNOS, eNOS, and p-AKT was significantly upregulated the VEGFA siRNA group (*P* < 0.05, Figures 6C,D). Decreased p-eNOS, eNOS and p-AKT expression in response to H19 silencing was also suppressed by VEGFA siRNA + miR29b inhibitor treatment (*P* < 0.05, Figures 6C,D).

## Discussion

Diabetes has become an increasingly serious global health problem. High blood glucose concentrations are an independent risk factor for the development and progression of AS (Bhupathiraju and Hu, 2016; Shah and Brownlee, 2016). Moreover, endothelial cell dysfunction is a leading factor in the development and progression of AS (Haybar et al., 2019). High blood glucose concentrations may induce damage in endothelial cells, mainly through the dual mechanisms of inflammation, and oxidative stress (Shi and Vanhoutte, 2017). However, the specific molecular mechanisms through which this could occur are unclear. Based on these data, we surmised that regulating vascular inflammation by regulating expression of inflammation-related genes may be a means of preventing and treating CVDs.

In recent years, many studies have confirmed the important role of ncRNA networks in the regulation of gene expression. Furthermore, interactions between lncRNA and miRNA are reportedly involved in a variety of physiological and pathological processes (Abdollahzadeh et al., 2019). One of the first identified lncRNAs, H19, plays a wide range of roles *in vivo*. H19 can function as both a tumor suppressor or oncogene and also as a regulator of growth and development in multiple mammalian embryo tissues (Bai et al., 2016; Yoshimura et al., 2018). A previous study revealed that H19 is up-regulated in AS and is related to AS progression (Pan, 2017), but the underlying regulation mechanism has not yet been determined. Overexpression of miR-222 protects against cardiac injury and miR-17-3p promotes cardiomyocyte proliferation and increased cardiomyocyte size through directly targeting TIMP3 (Wang et al., 2018). miR-29b is one of the first miRNAs that was found to be dysregulated in retinal cells and diabetic rats exposed to high glucose concentrations, suggesting that they are involved in the development of DM (Dantas da Costa E Silva et al., 2019). A recent *in vivo* study showed that miR-29b is involved in regulation of glucose balance and insulin secretion through a variety of target genes and influences the pathogenesis of diabetes (Dooley et al., 2016). The VEGF glycoprotein family has a strong ability to promote vascular endothelial cell division and proliferation and enhance capillary permeability. This family includes VEGFA, VEGFB, VEGFC, VEGFD, and the placenta growth factor. VEGFA has been demonstrated to show prominent activity with vascular endothelial cells (Tan et al., 2018) and VEGFA is a direct target gene for many miRNAs. For instance, miR-9 inhibits retinal neovascularization and tubule formation in diabetic retinitis and promotes apoptosis in retinal microvascular endothelial cells by targeting VEGFA (Liu, 2019). miR-150-5p may function as a tumor suppressor in colorectal cancer, making the miR-150-5p/VEGFA axis a potential therapeutic target in colorectal cancer treatment (Chen et al., 2018). miR-15a-5p acts as a regulator of *VEGFA* mRNA and of subsequent inflammation and fibrosis in peritoneal mesothelial cells (Shang et al., 2019). These data led us to further investigate the potential involvement of H19 miR-29b/VEGFA in the inflammatory response in individuals with DM.

In this study, we show that H19/miR-29b/VEGFA signaling pathway plays a role in hyperglycemic-induced endothelial dysfunction. We found that H19 expression is upregulated and miR-29b expression downregulated in ECs and blood samples obtained from individuals with DM. It has been widely reported that disease-associated lncRNAs and miRNA are detectable in the blood of patients (Yin et al., 2017; Zhang Y. et al., 2018; Yoffe et al., 2019). Therefore, lncRNA might present a potential biomarker for the dynamic monitoring of DM. In individuals with DM, H19 and miR-29b expression was consistent with the expression changes we observed in endothelial cells following glucose treatment for 48 h. VEGFA expression was consistent with H19 changes in the presence of high glucose concentrations. With increasing glucose concentrations in the growth medium of endothelial cells, H19, and VEGFA expression was significantly upregulated in endothelial cells, while miR-29b expression decreased in a dose-dependent manner. Furthermore, downregulation of H19 increases miR-29b expression and facilitates miR-29b-mediated VEGFA inhibition. The results of luciferase and RIP experiments suggest that H19 and miR-29b directly interact. Luciferase assay results revealed that miR-29b mimics decrease luciferase activity in wt-Luc-H19 transfected cells and that this effect is not observed in mu-Luc-H19-transfected cells. Exposure to high glucose concentrations increased wt-Luc-H19 luciferase activity and this effect was abrogated by the H19 mutant. We found that miR-29b mimics restrain luciferase activity in the wt-Luc-VEGFA reporter vector. However, this phenomenon was completely suppressed by mutation in the 3′-UTR of *VEGFA*. RIP experiments showed that the Ago2 protein was successfully precipitated from the digested cells by the Ago2 antibody. Both H19 and miR-29b were significantly enriched in the immunoprecipitates, suggesting that there is direct binding between H19 and miR-29b. These results indicate that H19 may in part regulate VEGFA expression by competing with miR-29b.

Next, we studied the link between H19/miR-29b/VEGFA and inflammatory factors in the pathogenesis of CVD by investigating TNF-α expression, ROS production, and NADPH oxidase activity. TNF-α, a prototype proinflammatory cytokine, increases endothelial permeability and disrupts endothelial integrity (Cui et al., 2018). ROS is considered to induce impairment of endothelial function in CVD. NADPH oxidase-derived ROS has been implicated in the activation of NF-κB and release of proinflammatory mediators (Yao et al., 2007). We identified significant inhibition of TNF-α expression, ROS production, and NADPH oxidase activity in H19 shRNA, VEGFA siRNA, and miR-29b mimic groups in the presence of high glucose. In addition, the miR-29b inhibitor reversed these effects. These results indicate that regulation of H19, miR-29b, and VEGFA expression modulates the expression of inflammatory factors that may contribute to the pathogenesis of CVD.

The AKT/eNOS signaling pathway is involved in key endothelial cell functions and in central cardiovascular regulation. To the best of our knowledge, the AKT/eNOS pathway plays an important role in improving endothelial function and alleviating AS (Xing et al., 2015). Our data show that phosphorylation of eNOS and AKT significantly increase in the presence of H19 shRNA and VEGFA siRNA and high glucose concentrations, and that these effects are reversed by the miR-29b inhibitor. Therefore, we speculate that H19 downregulates expression of VEGFA, the miR-29b target gene, and through competing with miR-29b. Moreover, modulation of H19/miR-29b/VEGFA may trigger up-regulation of the AKT/eNOS signaling pathway, indicating a potentially promising therapeutic strategy against AS.

We found a novel pathogenic link between lncRNA H19, miR-29b, and VEGFA in hyperglycemic-induced endothelial dysfunction. Suppression of H19 may alleviate inflammation in endothelial cells incubated at high glucose concentrations by upregulating miR-29b and inhibiting expression of VEGFA. These events are closely associated with activation of the AKT/eNOS signaling pathway, and can attenuate the progression of DM-related AS.

## Data Availability Statement

The data used to support the findings of this study are available from the corresponding authors upon request.

## Ethics Statement

This study protocols were approved by the Ethics Committee of Anhui Medical University.

## Author Contributions

H-QZ and HW contributed to the conception and design of the experiments, gave final approval, and agreed to be accountable for all aspects of work ensuring integrity and accuracy. X-WC and Z-FC drafted and critically revised the manuscript. X-WC, Z-FC, and Y-FW completed the human and cell experiments. X-WC, Z-FC, Y-FW, and QZ contributed to the analysis.

## Conflict of Interest

The authors declare that the research was conducted in the absence of any commercial or financial relationships that could be construed as a potential conflict of interest.

## Footnotes

1 http://www.targetscan.org/vert_71/
